# A method for mechanized hybrid rice seed production using female sterile rice

**DOI:** 10.1186/s12284-019-0296-8

**Published:** 2019-05-28

**Authors:** Yumei Xia, Ning Tang, Yuanyi Hu, Ding Li, Shuangcheng Li, Xiaolan Bu, Mulan Yu, Shaowu Qi, Yishan Yang, Hongjin Zhu, Chenying Cao, Ping Li, Longping Yuan, Mengliang Cao

**Affiliations:** 1 State Key Laboratory of Hybrid Rice, Longping Graduate School, Hunan University , Changsha, 410082, China/ Hunan Hybrid Rice Research Center, Changsha, 410125 China; 2 Collaborative Innovation Center, Hunan Agriculture University, Changsha, 410128 China; 3 Department of Biotechnology, Hunan Key Laboratory of Green Packaging and Application of Biological Nanotechnology, Hunan University of Technology, Zhuzhou, 412007 China; 4 Rice Research Institute, Sichuan Agricultural University, Chendu, 611130 China; 5 Southern Regional Collaborative Innovation Center for Grain and Oil Crops in China, Changsha, China

**Keywords:** Hybrid rice, Female sterility, Mechanization of hybrid seed production, Breeding

## Abstract

**Background:**

The breeding and large-scale adoption of hybrid rice is an important achievement in modern agriculture. Mechanized seed production is urgently needed for widespread adoption of hybrid rice because it can compensate for the shortage of manual labor to meet the growing food demands in China.

**Results:**

Here, we report the development of a mechanized hybrid rice seed production method using a female sterile rice. In this method, three closely linked gene expression cassettes were introduced into female sterile rice. The three expression cassettes are: 1) a rice female fertility gene expression cassette; 2) a pollen-lethal gene expression cassette; and 3) a red fluorescence protein gene expression cassette. During the self-fertilization process of a heterozygous transgenic rice plant, pollen grains carrying the transgene die off and cannot participate in fertilization; pollen grains not carrying a transgene can normally fertilize the female gamete, leading to fructification. By means of fluorescence-assisted sorting, homogeneous female sterile rice seeds are sorted out from other seeds carrying the transgene and are used for mechanized hybrid rice seed production; heterozygous seeds carrying the transgene can then be used in the multiplication of female sterile rice.

**Conclusions:**

This technology solves the difficulty of multiplying female-sterile rice, allows for mechanized production of hybrid rice seed, and will prove especially valuable in systems using a mixed-planting, mixed-harvesting approach. Moreover, it uses transgenic technology that has not yet been employed in a seed production process in which the output is non-transgenic seeds.

## Background

The breeding and large-scale adoption of hybrid rice is an important achievement in modern agriculture that contributes significantly to the global food supply. In China, the annual planting area of hybrid rice is about 16.7 million hm^2^, and area used for seed production is about 180,000 hm^2^ (Wang et al. 2013). Currently, the whole process of seed production is complicated, depends on manual labor, and has thus far been recalcitrant towards adoption of mechanization or automation of steps to improve efficiency. Urbanization, agricultural industrialization, and the implementation of the rural land circulation policy (Wang et al. 2011) have all contributed to a reduction in the rural population and available labor for rice production. Thus, labor-intensive seed production has become a powerful hindrance to popularization of hybrid rice. Mechanization of hybrid seed production could effectively decrease production costs and become a key element for widespread adoption of hybrid rice by farmers.

Currently, there are several methods available for mechanization of hybrid rice seed production: 1) Utilization of bentazon-sensitive and Basta-resistant genes (Fu et al. 2001; Wang et al. 2009; Zhang et al. 2008; Zhang, 2010; Zhu, 2010). This entails introduction of a bentazon-sensitive gene into the paternal line (restorer line) or a resistance gene against Basta herbicide into the maternal line (male sterile line). These transgenes will confer herbicide resistance for parental seeds and permit subsequent spraying of herbicide after pollination to remove the paternal line (restorer line) for mechanized harvesting. 2) Differences in parental seeds could potentially be exploited to use seed selectors for sorting (Maruyama et al. 1991; Lv et al. 1996; Tang et al. 2012; Wu et al. 2005; Yu et al. 2007), such as utilization of shell color to distinguish the maternal (male sterile) and paternal (restorer) lines (He et al. 2001; Li and Hu, 2010). The soundest method is to mechanically mix maternal line seeds with normal shell color and paternal line seeds before sowing, harvest all seeds together when they are mature, then use an automated color selector to sort and separate hybrid seeds. 3) Another semi-automated operation method. In this system, parents are planted intensively at different times. First, maternal and paternal samples are sown by artificial plantation, after pollination to remove paternal samples manually, and finally maternal samples are harvested with a machine (Gao et al. 2007; Zhu 2004).

Despite major successes, all of these systems have intrinsic drawbacks. At present, the mechanization of hybrid rice seed production, especially in mixed seed production, has not been able to achieve large-scale commercial applications in production.

The female sterile line is a promising genetic tool for use as the paternal line in hybrid seed production. In this system, female sterile restorers can pollinate male sterile lines to produce hybrid seeds. However, female sterile restorers do not produce self-pollinating seeds that affect the purity of hybrid seeds, making it unnecessary to remove them after pollination. The maternal (male sterile) and paternal (female sterile restorer) lines can therefore be mixed in production, thus improving the efficiency of cross-pollination under natural conditions (and the overall efficiency of hybrid seed production). This method will reduce labor input for seed production and will make hybrid seed production more conducive to mechanization (Gao et al. 2007).

In this study, we developed a system for reproduction of female sterile plants and explored its potential application in mechanization of hybrid seed production. To this end, we used the female fertility gene, *POLLEN TUBE BLOCKED 1* (*PTB1, LOC_Os05g05280*), which is essential for pollen tube growth and female fertility in rice (Li et al. 2013), coupled with a pollen-lethal gene and a red fluorescence protein gene.

## Results

### Construction of a Female Sterility System Using *PTB1*

**Fig. 1:**
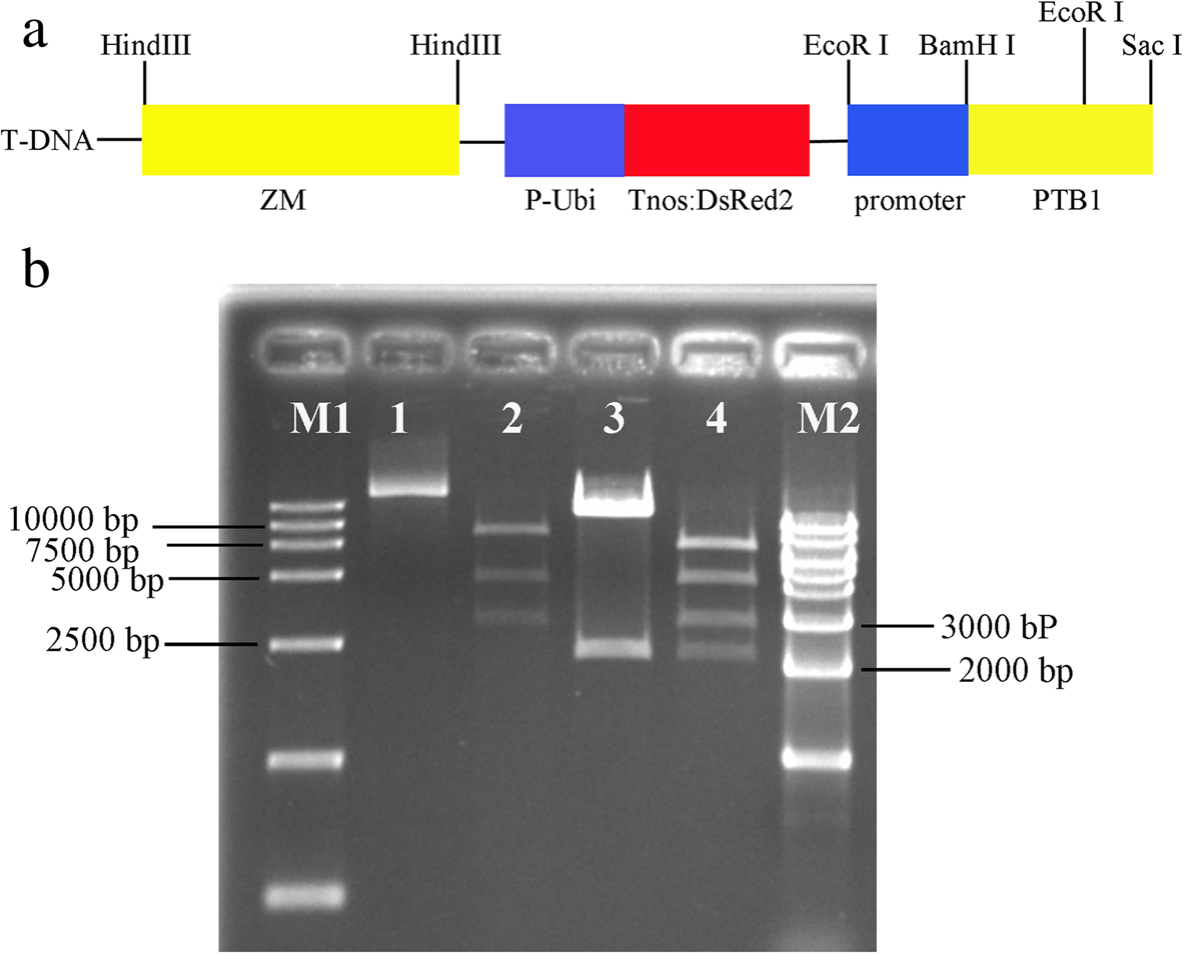
Construction of the *pFS4* vector. **a** Three expression cassettes in the T-DNA. Pollen lethal gene cassette was about 4.7 Kb; the *DsRed* fluorescent bioreporter gene cassette was about 3 Kb; and a rice female fertility gene cassette was about 3 Kb. **b** Identification of *pFS4* by restriction digest. M1-DL15000 marker; lane 1, *pFS4* plasmid; lanes 2–4, *pLZM* plasmid, *pHBA* plasmid, and *pFS4* plasmid were double digested by *EcoR*I and *Hind*III, respectively. *pLZM* vector, constructed in our laboratory, contained *DsRed* and *ZM*; M2–1 Kb marker

We constructed the *pFS4* vector for *A. tumefaciens* (Fig. 1a) with three expression cassettes in the T-DNA: 1) a rice female fertility gene cassette; 2) a pollen lethal gene cassette; and 3) the *DsRed* fluorescent bioreporter gene cassette. Double digestion of plasmid *pLZM* and *pFS4* by *EcoR*I and *Hind*III produced one fragment, *ZM*, of ~ 4.7 Kb*,* and another ~ 3.0 Kb fragment, *DsRed.* Double digestion of plasmids *pHBA* and *pFS*4 by *EcoR*I and *Hind*III produced a ~ 2.5 Kb fragment, *PTB1*. These results showed successful construction of the T-DNA insert (Fig. 1b).

### Agrobacterium-Mediated Transformation and Recovery of Transgenic Rice Plantlets

**Fig. 2:**
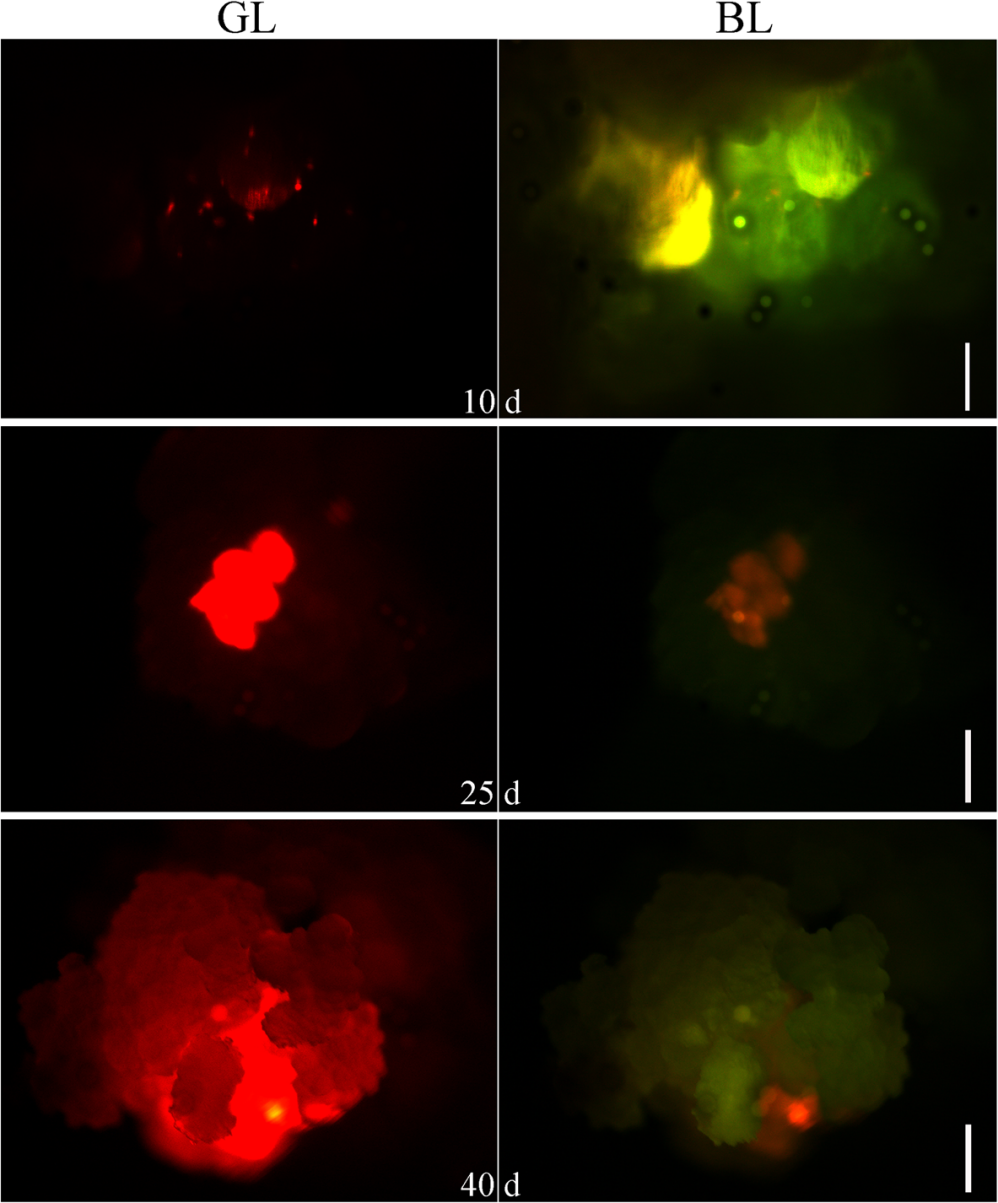
Transgenic callus after different incubation periods under a green light and blue light, respectively. Green light (GL) and blue light (BL). (Scale bars, 1.0 mm)

We transformed MingHui86(MH86) with the *pFS4* vector using the *Agrobacterium*-mediated method described above. We screened antibiotic resistant calli by red fluorescence (Fig. 2); calli displayed point-like fluorescence on the 10th day after transfer. By the 40th day of growth, a large portion of each callus displayed red fluorescence. The calli expressing RFP protein produced an increasingly strong red fluorescence signal with prolonged incubation. This result showed that only part of each callus was expressing the *DsRed* gene cassette, thus indicating that the red fluorescence signals were derived from stable transformation and integration of the transgene and not from transient expression. Red fluorescent calli were transferred 40 days later to regeneration medium for further induction of expression cassettes and tissue differentiation.

Out of 2440 calli, 188 antibiotic resistant calli were transferred to differentiation medium. We obtained 25 transgenic plantlets from 188 antibiotic resistant calli. Due to the presence of chimeric cells during transformation, not all of the transgenic plantlets exhibited red fluorescence (Table 1).

**Table 1:** Screen of transformed MingHui86 with the *pFS4* vector

batch	number of calli	number of antibiotic resistent calli	number of transgenic plantlets	number of positive transgenic plantlets (RFP)	number of positive transgenic plantlets (PCR)	rate of positive transgenic plantlets
first	840	65	9	6	6	0.71%
second	880	73	9	6	6	0.68%
third	720	50	7	5	5	0.69%

Note: rate of positive transgenic plantlets is (number of positive transgenic plantlets (RFP)/number of calli) × 100

### Molecular Identification of Transgenic Plantlets

**Fig. 3:**
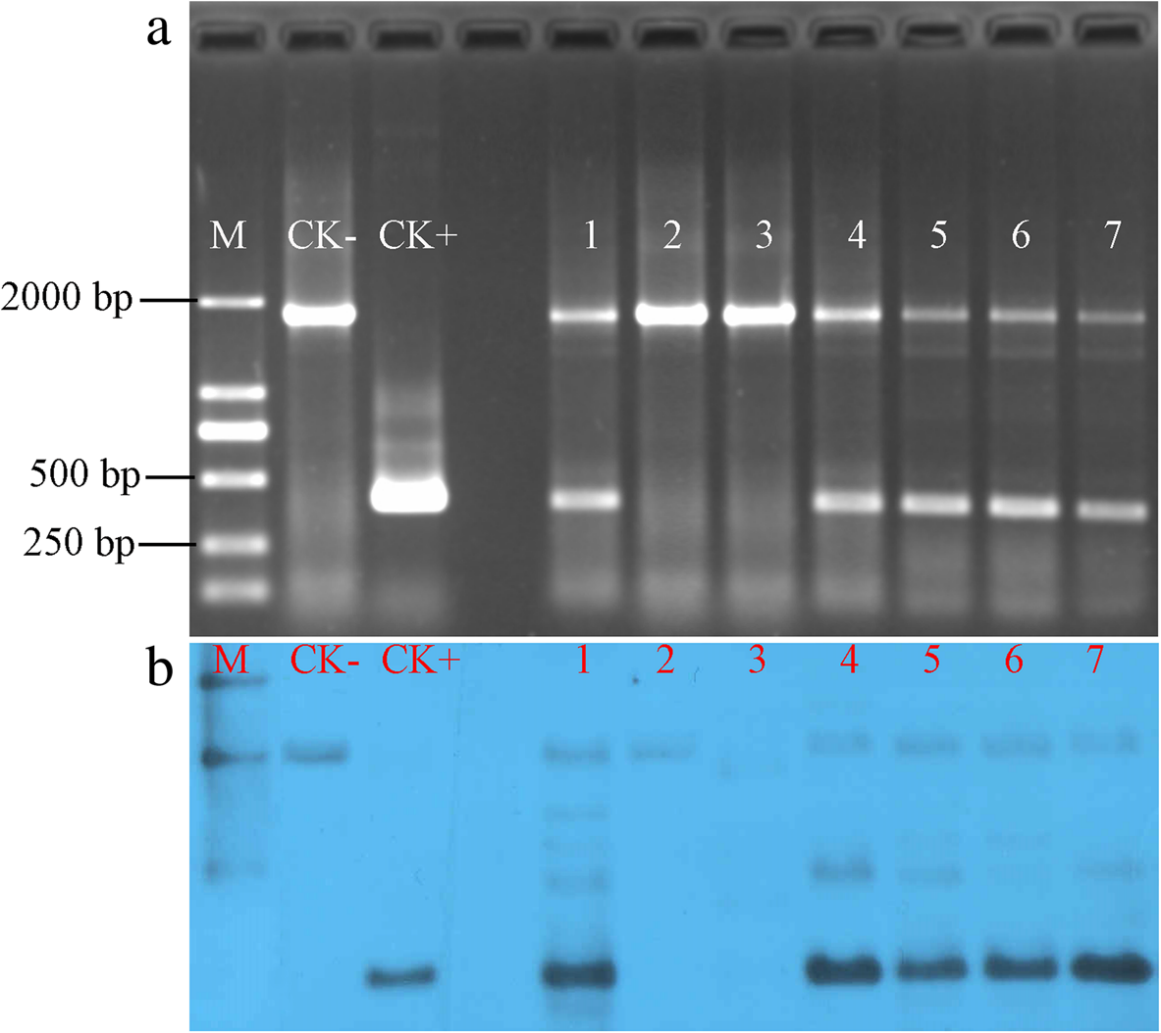
Molecular identification of transformants. **a** PCR analysis of T_0_ transformants shows the presence of the 1761 bp *PTB1* gene in the rice genome (CK-), 412 bp fragment of *PTB1* CDS in the *pFS4* plasmid (CK+), and both 1761 bp and 412 bp fragments in T_0_ transgenic plants. **b** Southern blot analysis to confirm insertion of T-DNA in transformants. Genomic DNA from a non-transgenic plant (CK-), the *pFS4* plasmid (CK+), and T_0_ generation transformants (1–7) were digested with *EcoR*I and hybridized with the *PTB1* CDS probe. Southern blots show that *PTB1* CDS sequence was stably integrated into the genomes of transgenic lines

All transgenic plantlets were selected for molecular analysis to check for successful transformation. PCR screens were initially used to test the putative transformants for the presence of the *PTB1* CDS using genomic DNA template extracted from T_0_ transgenic plantlets with the *PTB1- F2/PTB1 -R2* primer set. All T_0_ transgenic plantlets that carried the red fluorescence cassette showed successful transformation with *PTB1* CDS (Fig. 3a). Southern hybridization analysis was performed on the T_0_ transgenic plantlets using a 0.4 kb CDS sequence of *PTB1* as the probe. All T_0_ transformed plantlets that exhibited red fluorescence also showed strong hybridization signals (Fig. 3b), which was consistent with the initial PCR detection. The copy numbers of the *PTB1* gene integrated into the MH86 for the transgenic lines were 1~4. These results demonstrated that the T-DNA inserts contained all three functional modules, *PTB1, DsRed,* and *ZM-AA1*, and that they were successfully integrated into the rice genome. This screen also showed that the red fluorescence protein (*DsRed*) gene is an efficient marker for transgenic plants.

### Phenotype of the Nuclear Female Sterility System

The T_0_ transgenic plants were cross-pollinated with the *ptb1* mutant plant described above. Since *PTB1* is a sporophytic female fertility gene, a hemizygous *PTB1* transgene in the *ptb1* mutant plant can fully restore female fertility. *ZM-AA1* driven by a *PG47* promoter is a gametophytic factor that disrupts starch accumulation only in the transgenic pollen (Allen and Lonsdale, 1993), resulting in deactivation of only the transgenic pollen grains produced by the hemizygous transgenic plant.

**Fig. 4:**
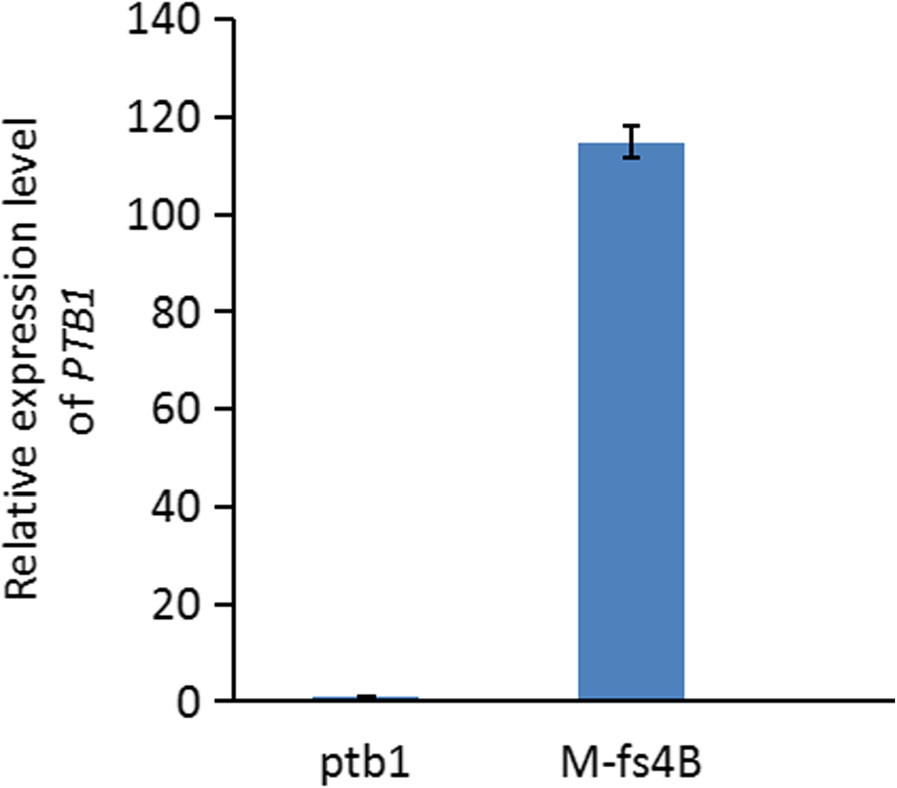
Differential expression level of the *PTB1* gene between the transgenic line M-fs4B and *ptb1* mutant. The relative expression level of the *PTB1* gene was determined using q-PCR analysis with RNA isolated from the pistil of the transgenic line M-fs4B and *ptb1* mutant. Relative expression levels were measured as the mean ± s.e.m. (*n* = 3). All P values are based on two-tailed *t*-tests

One transgenic plant, M-fs4B, where the *PTB1* gene was introduced in the *ptb1* background, was selected for further study. q-PCR results demonstrated that the relative expression level of the *PTB1* gene in the pistil of M-fs4B was significantly higher than that in the *ptb1* mutant (Fig. 4).

**Fig. 5:**
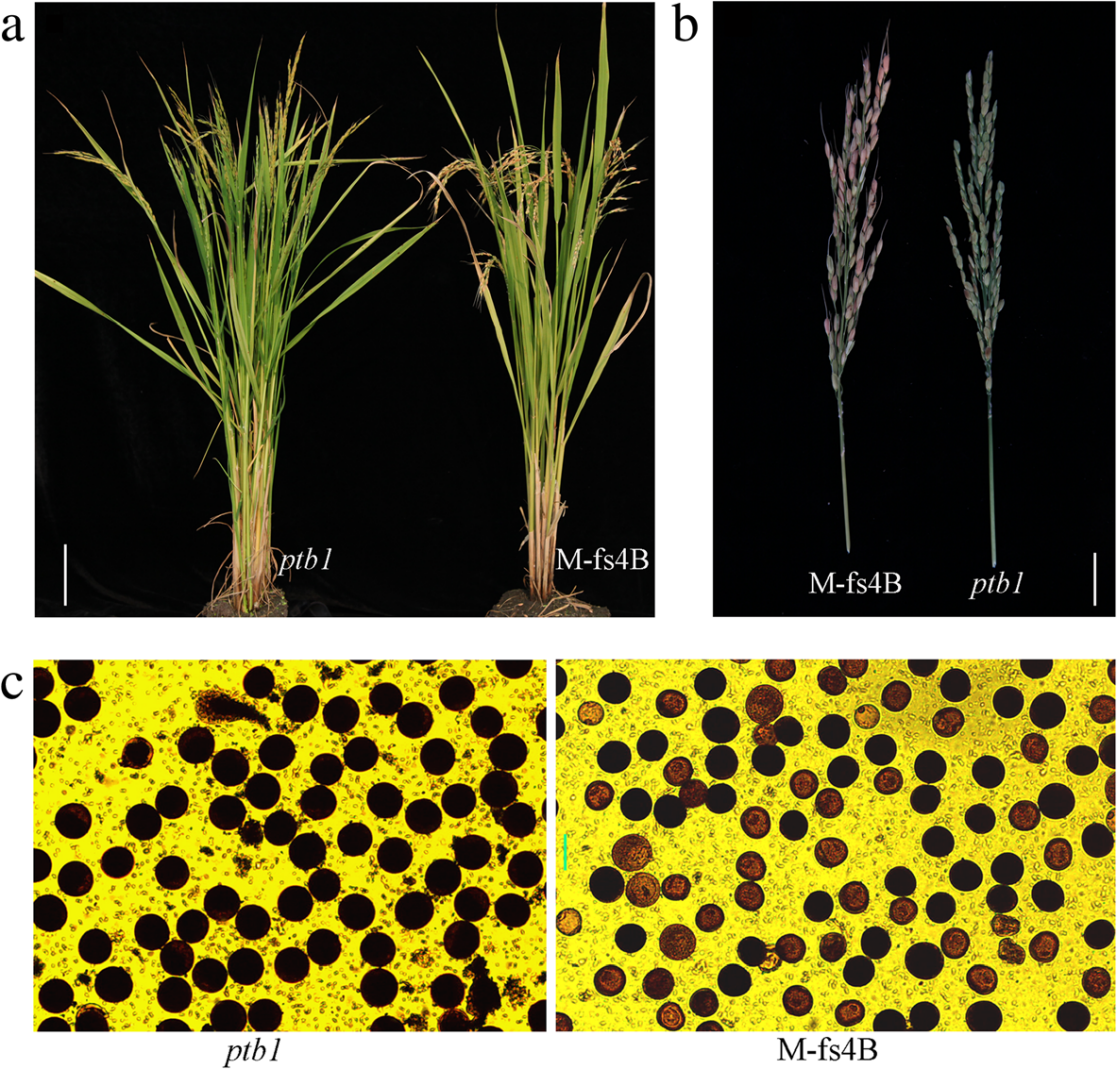
Phenotype of the nuclear female sterility system. **a** Transgenic M-fs4B and *ptb1* mutant plants. (Scale bar, 12.5 cm) (**b**) M-fs4B and *ptb1* panicles. (Scale bar, 3.4 cm.) (**c**) I_2_-KI staining of M-fs4B and *ptb1* pollen grains. (Scale bar, 40 μm)

The plant M-fs4B exhibited normal vegetative and reproductive growth (Fig. 5a), a 1:1 ratio of fluorescent seeds (with a hemizygous transgene) to non-fluorescent seeds (no transgene) in the panicle (Fig. 5b), and a 1:1 ratio of fertile to defective pollen grains (Fig. 5c). Furthermore, eight yield-related agronomic traits, including plant height (PH), panicle number (PN), panicle length (PL), flag leaf length (FLL), flag leaf width (FLW), spikelet number (SN), seed setting rate (SSR), and ratio of fluorescence seeds (RFS) were investigated, and compared between M-fs4B and the *ptb1* mutant. Except for SSR and RFS, no significant differences were observed between M-fs4B and the *ptb1* mutant. The SSR of M-fs4B plant reached up to 82.16%, of which 52.23% were fluorescent seeds (Table 2).

**Table 2:** Comparison of main agronomic traits between transgenic line M-fs4B and the *ptb1* mutant

Sample	PH (cm)	PN	PL (cm)	FLL (cm)	FLW (cm)	SN	SSR (%)	RFS (%)
M-fs4B	110.44 ± 3.80	8.0 ± 1.58	25.94 ± 1.46	47.34 ± 2.30	1.58 ± 0.07	168.2 ± 14.53	82.16 ± 2.46	52.23 ± 5.49
*ptb1*	113.4 ± 3.01	7.4 ± 1.14	26.14 ± 1.28	46.74 ± 2.58	1.60 ± 0.07	157.8 ± 8.89	0.71 ± 0.20	0
*P*-value	0.21	0.51	0.82	0.71	0.75	0.21	1.25E-12	2.44E-08

**Fig. 6:**
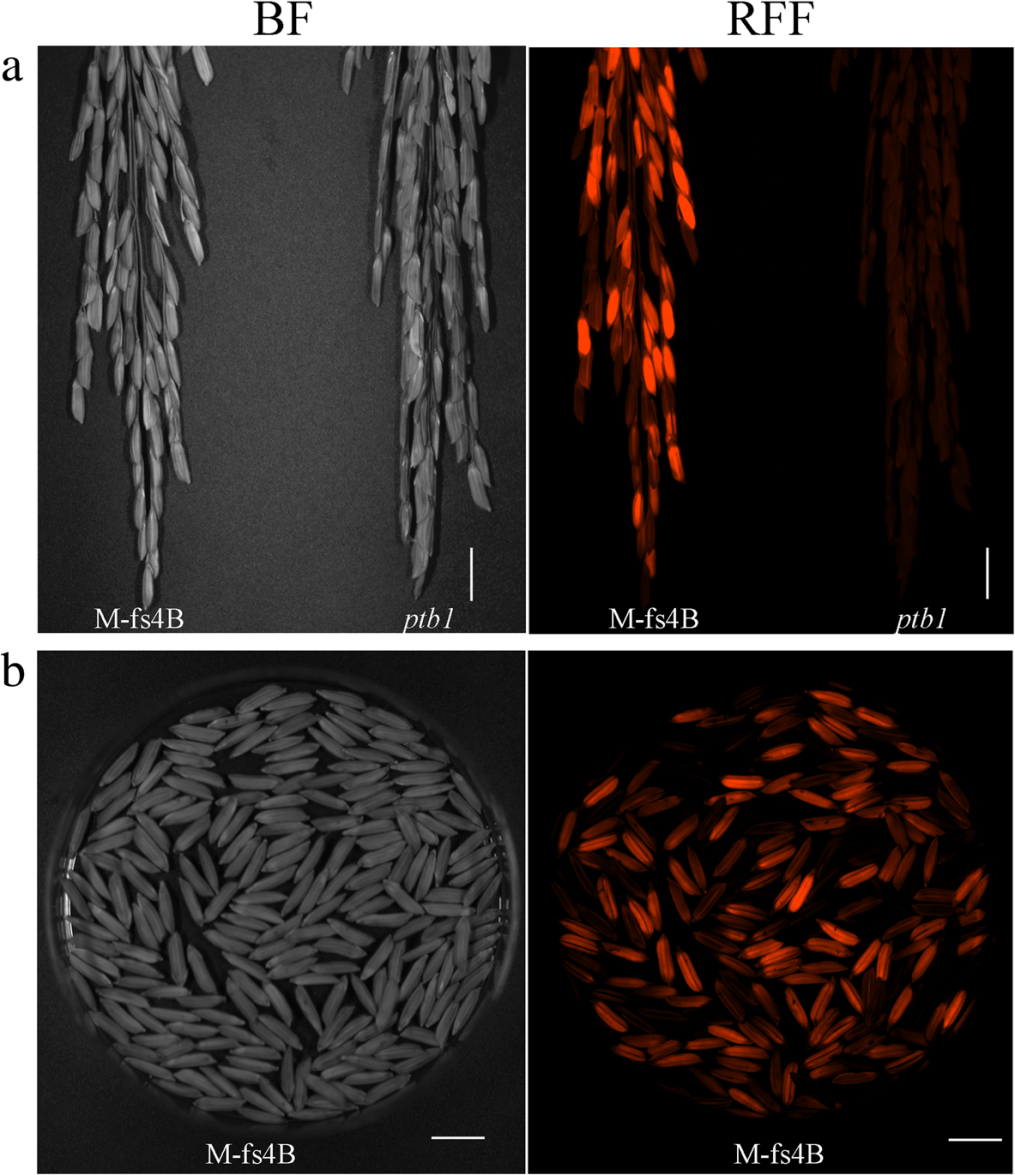
(**a**) M-fs4B and *ptb1* panicles under bright field (BF) and a red fluorescence filter (RFF). (Scale bar, 1.3 cm). (**b**) Seeds of M-fs4B under bright field (BF) and red fluorescence filter (RFF). (Scale bar, 1.0 cm)

The seeds were sorted out manually based on the fluorescence (Fig. 6a, b) and cultivated for the next generation. All plants from the fluorescent seeds were fertile and genetically identical to the parent *ptb*1, while all plants from the non-fluorescent seeds were female sterile. The fluorescence screening and fertility examination experiments were repeated for five generations, with a total of more than 1 million seeds, and all showed the same results, indicating the transgene was stable.

### M-fs4B is Promising for Mechanized Commercial Seed Production of Hybrid Rice

**Fig. 7:**
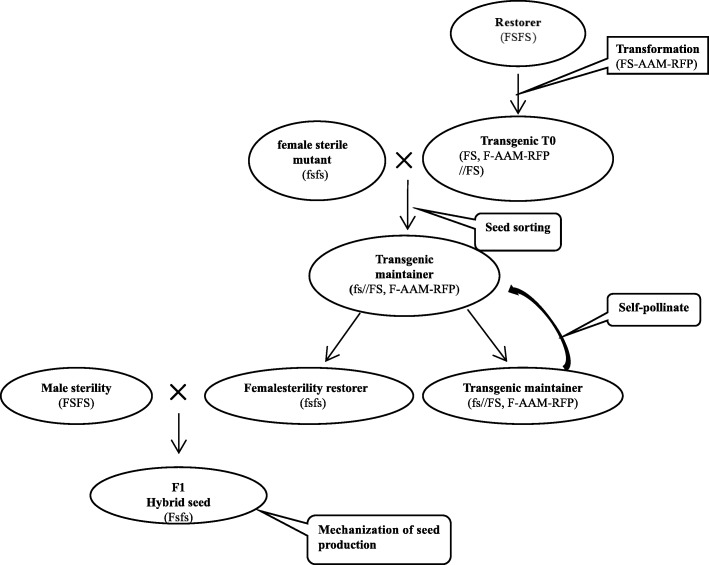
Model of a nuclear female sterility system for mechanization of hybrid seed production in rice. The restorer line was created by transformation of wild-type commercial line MH86 with a vector construct containing three transgene expression cassettes: (i) the WT fertility gene (FS) to restore the female fertility, (ii) the α-amylase gene (AAM) to devitalize transgenic pollens, and (iii) the red fluorescence protein (RFP) gene to distinguish the transgenic seeds from the non-transgenic seeds. A hemizygous transgene in the female sterile mutant plant can fully restore the female fertility since the female fertility gene is a recessive sporophytic gene. The α-amylase gene, driven by a pollen-specific promoter, disrupts starch accumulation only in the transgenic pollen so only the transgenic pollen grains produced by the hemizygous transgenic plant are defective, while the non-transgenic pollen grains are viable for pollination. The resulting transgenic maintainer plant (FS, FS-AAM-RFP//fs) produces male gametes (MG) of one genotype (fs) and female gametes (FG) of two genotypes (fs and FS, FS-AAM-RFP). Self-pollination of the transgenic maintainer line generates the transgenic seed (FS, FS-AA-RFP//fs) and female sterile seed (fs//fs) in a 1:1 ratio, and the seeds can be sorted based on red fluorescence. The female sterile seeds can be propagated via self-pollination. The female sterile plants (paternal line) can be pollinated with male sterile plants (maternal line) for mechanized production of hybrid seeds. The restorer was transformed with vector *pFS4*

To determine the potential of M-fs4B as a maintainer line, propagation of M-fs4B seeds was achieved by M-fs4B plant self-pollination, with the maintainer line seeds and female sterile seeds sorted using red fluorescence. Female sterile plants (M-fs4A) can be cross-pollinated with male sterile plants for mechanized seed production of hybrid rice. We employed the strategy shown in Fig. 7 to develop mechanized production of seeds of hybrid rice using the transgenic *ptb*1 mutant line because the *ptb1* mutant exhibited desirable agronomic traits.

During the self-fertilization process of a heterozygous transgenic rice plant, pollen grains carrying the transgene die off and cannot participate in fertilization; pollen grains not carrying a transgene can normally fertilize the female gamete, leading to fructification. By means of photoelectric selection, homogeneous female-sterile rice seeds can be obtained and used for mechanized seed production; heterozygous seeds carrying the transgene continue to be used in the multiplication of female-sterile rice.

Although the technology involves transgenics, only the maintainer line carries the transgene. Both the female sterile seeds and hybrid seeds are non-transgenic. Thus, transgenic oversight is applicable only to cultivation of the maintainer line, which requires only minimal acreage, while production of hybrid seeds and hybrid cultivation do not require transgenic oversight. Application of this technology will greatly enhance hybrid rice breeding and mechanized production.

## Discussion

Traditional hybrid rice production is complicated and depends on manual labor, while mechanized production methods are difficult to introduce. Improvement to production output and quality are only achieved through careful cultivation and farming techniques. Due to urbanization, agricultural industrialization, and the implementation of the rural land circulation policy, rural populations in China will further decrease, and traditional production methods have already fallen out of sync with trends in development. Hybrid rice cultivators should take advantage of many kinds of biological resources and breed strong hybrid combinations to adapt to current mechanized production demands.

Wang et al. (2019) and Khanday et al. (2019) developed systems to enable self-propagation of F1 hybrids. Both of their systems resulted in asexual seeds, and made progress in apomixis in hybrid rice. In the system of Wang et al., the panicle seed setting rate was significantly reduced to ~ 4.5%. In the system of Khanday et al., they obtained haploid mother plants at frequencies of 26% and 15%. Though both of their systems enable self-propagation of F1 hybrids, they are not really applicable in the production of hybrid rice. Only the haploid mother plants at frequencies of 100% can really achieve apomixis in hybrid rice. The propagation of F1 hybrid rice still needs traditional methods, because it is complicated and depends on manual labor.

Nuclear female sterile mutants are common in flowering plants and using female sterility as a trait for mechanization of hybrid rice production may be a profitable avenue to pursue. However, commercial application of these mutants is limited because of the difficulty in propagation of pure female sterile lines.

Using female sterile genes for mechanized seed production technology is the newest direction in seed production research. This technology reduces the difficulty of multiplying female-sterile rice and allows for mechanized seed production for hybrid rice and, of particular interest, for mechanized seed production using a mixed-planting, mixed harvesting approach. Moreover, this method uses transgenic technology, which has not yet been deployed for the seed production processes notwithstanding that the seeds under production are non-transgenic. Application of this technology will facilitate mechanization production of hybrid rice seeds and greatly reduce costs for hybrid rice seed production.

A method for seed production for hybrid rice was developed by transformation of a male sterile mutant with a fertility restoration gene linked to the α-amylase gene (Chang et al. 2016). Our female sterility system combined with that male sterility system can together form the genetic tool needed for third-generation hybrid rice. The third-generation of hybrid rice can achieve mechanized seed production on the basis of stable fertility and free combination, to improve the economic benefits of breeding and the reproduction industry.

## Conclusions

In this work, we deployed the *PTB1* gene as the female fertile gene, maize α-amylase as the pollen-lethality gene, and *DsRed* as the seed-marker gene. A transgenic line, M-fs4B, was identified and was introduced into the *PTB1* gene, the pollen-lethality gene, and *DsRed* gene in the *ptb1* background. As expected, self-pollination of M-fs4B propagated itself and the female sterile M-fs4A in 1:1 ratio. The availability of a sorting machine makes it possible to separate the transgenic maintainer seeds from the non-transgenic female sterile seeds based on the presence of red fluorescence, which ensures high purity of both lines at a commercial scale. Female sterile plants (M-fs4A) can be cross-pollinated with male sterile line for mechanized seed production of hybrid rice, indicating that the system is promising for widespread use.

## Materials and Methods

### Plant Materials and Growth Conditions

The *indica* rice (*Oryza sativa*) variety MingHui86 (MH86) is a widely used *indica* restorer line and is relatively easy to transform as an *indica*, so it was used as a transgenic acceptor in this study. The *indica* cultivar mutant *ptb1* (Ping Li Lab, Sichuan Agricultural University), a female sterile line obtained from a spontaneous mutation in an *indica* cultivar (Li et al. 2013), was used for backcrossing. All of the plants were grown in a paddy field in the cities of Changsha and SanYa during the natural growing seasons and maintained regularly.

### Plasmid Construction

To develop the female sterile system for mechanized hybrid rice seed production, the *pFS4* vector was constructed. *pFS4* contained three expression cassettes: 1) a rice female fertility gene expression cassette carrying the *PTB1* gene, which encodes a ring-type E3 ubiquitin ligase and promotes pollen tube (PT) growth by controlling the amount and depth of growth of PTs in the rice reproductive tract, under its native promoter to restore female fertility (Li et al. 2013); 2) a pollen-lethal gene expression cassette, the maize α-amylase gene *ZM-AA1* with an amyloplast-targeting signal peptide from the maize *brittle-1* gene (*Bt1*) (Chang et al. 2016; Sullivan et al. 1991), under a *PG47* promoter (a pollen-specific promoter) (Allen and Lonsdale, 1993; Wu et al. 2016) to devitalize transgenic pollen; and 3) a fluorescent bioreporter gene expression cassette using the Red Fluorescence Protein gene *DsRed* (Matz et al. 1999) under a *35S* enhancer and *LTP2* promoter (an aleurone-specific promoter) (Kalla et al. 1994; Albertsen et al. 2006) for detection of the transgenic seeds.

First, the fragment containing multiple cloning sites between *EcoR*I and *Sal*I was removed by restriction digest from *pCAMBIA1300* (CAMBIA, Canberra, Australia), and was then cloned into another *pCAMBIA1300* between *EcoR*I and *Xho*I, resulting in the construct *pFS1*. Second, the fragment containing the terminator-*DsRed*-promoter was digested from vector *pLJ02* (Shaohong Qu Lab, Zhejiang Agricultural Academy) and ligated into *pFS1* between *EcoR*I and *Hind*III, generating *pFS2*. Third, the *PTB1* CDS and promoter fragment, in which the *Hind*III site was previously closed, were digested from the vector *PHB-PPTB1-PTB1* (Ping Li Lab, Sichuan Agricultural University) and then cloned into *pFS2* between *EcoR*I and *Sac*I, generating *pFS3*. Finally, the terminator**-***ZM-AA1-*promoter fragment was synthesized and then cloned into *pFS3*, forming *pFS4* .

### Agrobacterium Transformation and Regeneration

The *pFS4* construct was introduced into *Agrobacterium tumefaciens* EHA105 and transformed into the *indica* rice line MH86. Rice embryos were sterilized and then cultured on N6B5 basal medium. The calli were transferred to fresh N6B5 basal medium following 10 days of growth and were then incubated for 4 more days. The calli and *pFS4-*carrying *Agrobacterium* were suspended in liquid acetosyringone (AS) medium for 20 min at 28 °C. After transformation, the calli were separated and incubated on solid AS basal medium for 3 days at 28 °C in the dark. Following this incubation, calli were transferred to a selective medium and incubated in the dark at 28 °C for 10–15 days. Antibiotic resistant fluorescent calli were screened with a Leica MZ16FA microscope (Fig. 2).

After 40 days of incubation, antibiotic resistant fluorescent calli were transferred to differentiation medium and cultured at 28 °C under continuous light conditions for 2–4 weeks. After 1–2 weeks of culture on differentiation medium, antibiotic resistant calli produced green spots that were subsequently grown into shoots after another 2 weeks of incubation. Finally, transformants were then positioned on rooting medium, within 2–3 weeks of the development of multiple tillers. The plantlets were transferred into pots with soil in a green house. All of the media were produced by our laboratory and all chemicals were purchased from GuoYao.

### Transgene Determination

PCR amplification screens were used to confirm the presence of T-DNA cassettes and to ascertain their genetic linkage in transgenic plants. Total genomic DNA was extracted from leaf tissue of both transformed and untransformed plants using the CTAB pyrolysis method (Long et al. 2018). The PCR analysis was carried out using 2 × EasyTaq PCR SuperMix (TransGen). The *PTB1* CDS sequence was amplified with specific primers PTB1-F2 (5′-TTATTGTCTTGTCGGTTCTTGTGCT-3′) and PTB1-R2 5′-AACTTCGGGAGTTCTTGAATCAGTG − 3′).

For Southern blot analysis, total genomic DNA from rice leaves was prepared using the method cited above. Purified DNA for each sample, digested with *EcoR*I was electrophoresed on 1% (w/v) agarose gel, then transferred onto Hybond-N_+_ nylon membranes (Amersham, Germany), and fixed with a UV Crosslinker (Fisher Biotech, USA) set to deliver an energy dosage of 700 J m^− 2^ as recommended by the manufacturer. These blots were hybridized with a *PTB1* specific probe labeled with [a-32P] dCTP (Amersham, Germany). The membrane was photographed using X-ray film (Kodak, USA).

### Expression Analysis

Total RNA was extracted from the pistil using TRIzol reagent (Invitrogen) and reverse transcribed using the TransScript One Step gDNA Removal and cDNA Synthesis SuperMix kit (YEASEN); RT-PCR was then performed according to the manufacturer’s instructions. q-PCR was carried out in a total volume of 20 μl containing 4 μl cDNA, 2 μl gene-specific primers (2 μM), 10 μl 2 × SYBR Green Mix (YEASEN) and 2 μl ddH_2_O using an AB GeneAmp PCR System according to the manufacturer’s instructions. Measurements were obtained using the relative quantification method (Livak and Schmittgen, 2001). The rice *Actin1* gene was used as the internal control. Each measurement was determined for at least two biological samples and using three replicates for each sample.

### Microscopy and Assessment of Transgenic Traits

Plants and organs were photographed with a Canon EOS5D MarkIII digital camera and Nerrton 7.0 Bio imaging system (VILBERLOURMAT), respectively. To analyze pollen fertility, pollen grains at the anther dehiscence stage were released and then stained with I_2_-KI solution before photography using a LEICA DM500 microscope.

We choosed the One copy number T_0_ transgenic line for *PTB1* gene as the female parent and backcrossed with a *ptb1* mutant (paternal line) described above, and the resulting fluorescent and non-fluorescent F_1_ seeds were sorted manually using DFP-1™ Dual Fluorescent Protein Flashlight (NIGHTSEA) to illuminate the red florescence. F_1_ fluorescent hybrid plants (maternal line) were backcrossed with indica rice *ptb*1 (paternal line) and the fluorescent and non-fluorescent F_2_ seeds were sorted manually using DFP-1™ Dual Fluorescent Protein Flashlight (NIGHTSEA) to illuminate the red florescence. F_2_ fluorescent hybrid plants were repeatedly backcrossed with *indica* rice *ptb1*. After four generations, a stable fluorescent hybrid rice identical to the parent *ptb1* was obtained and the ratio of fluorescent progeny was tracked. The pollen fertility of fluorescent plants were analyzed by I_2_-KI staining as described above (Fig. 5c).

### Investigation and Comparison of Yield-Related Agronomic Traits

An array of morphological characters of the transgenic line M-fs4B and *ptb1* mutant, including PH, PN, PL, FLL, FLW, SN, SSR, and RFS, were investigated at Changsha, Hunan province, in 2018. PH was measured as the distance from ground to the top of main panicle. PN was counted as the number of tillers with normal vegetative and reproductive growth. PL was the length of main panicle measured in the maturity stage. FLL and FLW were respectively the length and the widest part of the flag leaf. SN was counted as the total number of spikelets of the main panicle in the maturity stage. SSR was estimated as the number of full grains divided by the total grains, including the empty grains. RFS was measured as the percentage of fluorescent seeds divided by all the full grains. All the traits were measured as the mean ± s.e.m. (*n* = 5). All *P* values are based on two-tailed *t*-tests in Excel software.

## Abbreviations

BF: Bright Field
BL: Blue Light
CDS: Coding Sequence
CK: Control Check
cm: Centimeter
CTAB: Cetyl Trimethylammonium Bromide
DNA: Deoxyribonucleic Acid
FLL: Flag Leaf Length
FLW: Flag Leaf Width
GL: Green Light
Kb: Kilobase
MH86: MingHui86
mm: Millimeter
PCR: Polymerase Chain Reaction
PH: Plant Height
PL: Panicle Length
PN: Panicle Number
RFF: Red Fluorescence Filter
RFP: Red Fluorescence Protein
RFS: Ratio of Fluorescence Seeds
SN: Spikelet Number
SSR: Seed Setting Rate
μm: Micron
